# Infrared Camera Geometric Calibration: A Review and a Precise Thermal Radiation Checkerboard Target

**DOI:** 10.3390/s23073479

**Published:** 2023-03-26

**Authors:** Ahmed ElSheikh, Bassam A. Abu-Nabah, Mohammad O. Hamdan, Gui-Yun Tian

**Affiliations:** 1 Department of Mechanical Engineering, American University of Sharjah, Sharjah 26666, United Arab Emirates; b00070833@alumni.aus.edu (A.E.); mhamdan@aus.edu (M.O.H.); 2 School of Engineering, Newcastle University, Newcastle upon Tyne NE1 7RU, UK; g.y.tian@newcastle.ac.uk

**Keywords:** lens distortion, geometric calibration, thermography, infrared

## Abstract

Different infrared (IR) planar geometric calibration targets have been developed over the years that exploit a well-established and flexible optical camera geometric calibration procedure following the pinhole approximation. This geometric calibration is typically neglected in IR cameras, due to the relatively low resolution of thermal images and the complex IR targets needed for the geometric calibration in comparison to the optical targets. In this study, a thorough literature review of numerous IR camera geometric calibration targets, along with their respective outcomes, were summarized and leveraged to deliver a practical checkerboard target for less experienced end users, while offering the lowest reprojection errors. It was concluded that the fabrication of high emissivity contrast and precise square points of intersection within a checkerboard pattern extends the accuracy of capturing these control points in a thermal image for an optimized IR camera geometric calibration. Accordingly, two simple planar checkerboard targets were fabricated using laser engraving and ultraviolet (UV) printing technologies on a polished stainless steel (SS304) plate. The UV-printed checkerboard target on a polished metallic alloy delivered the lowest mean reprojection error (MRE) of 0.057 pixels and the lowest root mean square error (RMSE) of reprojection of 0.063 pixels, with a standard deviation lower than 0.003 pixels. The UV-printed design offers better accuracy than any other checkerboard calibration target, and comparable results to the best prominent circular pattern results reported in the literature.

## 1. Introduction

Infrared thermography (IRT) is the practice of capturing thermal images using infrared (IR) cameras by measuring the infrared radiation emitted by objects. IR radiation is constantly emitted from objects at temperatures above absolute zero, where the intensity and wavelength of IR radiation mainly depend on the object’s surface temperature [1,2,3]. To guarantee the accurate temperature recording and measurement of the object’s surface, thermographic or IR sensors require proper thermal calibration [4]. The thermal calibration of IR cameras is typically based on blackbody simulators [1] or thermocouple systems [5], which produce controlled thermal conditions to calibrate and ensure accurate thermal recordings by the IR camera. Apart from providing information on the thermal condition of a particular scene or object, IR cameras bear the feature of providing geometrical information on the object being imaged [6]. The geometrical information could be utilized to size objects in a particular scene, measure distances in real-world coordinates, or in image reconstruction by aligning different images of the same scene [6]. Nondestructive testing and evaluation (NDT&E) of material, involving the use of IR cameras for crack size estimation using vibrothermography [7,8,9,10] or eddy current thermography [11,12] and thermal diffusivity estimation in metallic components [13], requires accuracy in the thermal mapping to the world coordinate geometry within the IR camera field of view (FOV). To accurately retrieve geometrical information of imaged objects, distortions introduced by the natural curvature of the IR camera’s lens should be removed through geometrical calibration. However, geometrical information is typically neglected in IRT due to two main reasons, namely, IR cameras provide relatively low-resolution images, and a complex geometrical calibration procedure is needed to extract geometrical information when compared to visible cameras [6]. Thus, IRT applications generally involve the thermal imaging and the analysis of a particular body, avoiding any geometrical information [14,15].

**Figure 1:**
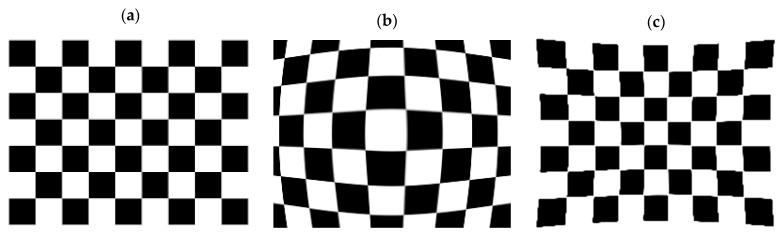
A schematic representation of a typical checkerboard with (**a**) no distortion, (**b**) barrel distortion and (**c**) pincushion distortion.

Geometric calibration is the process of imaging a calibration target with geometrically known features to determine the intrinsic and extrinsic parameters of a camera. Intrinsic parameters are values of the interior orientation elements of a camera, such as the principal points in image coordinate systems, focal length, skewness and, most importantly the radial and tangential distortions caused by the lens [16,17,18]. Radial and tangential distortions caused by the camera’s lens disfigure the captured images by introducing barrel or pincushion effects [19], as displayed in Figure 1. Extrinsic parameters, however, allow the transformation of a 3D geometrical scene to the camera’s 3D coordinate frame. This permits the representation of the spatial location of the camera in a 3D scene, allowing for measurements in world coordinates [6]. Thus, geometric calibration is crucial in photogrammetry to determine the relationship between 3D world coordinate points and 2D image coordinate points on the detector level of the camera [19]. Visible camera calibration is usually performed using the widely known checkerboard pattern that is printed using inexpensive off-the-shelf printers [20,21,22,23]. IR cameras do not display the visible color of an imaged object, since visible and IR cameras operate at different spectrum bands. Thus, directly employing the typical paper-printed checkerboard pattern used to calibrate visible cameras to IR cameras is not suitable, as the features of the checkerboard pattern are invisible at normal temperature conditions [24]. This causes the contrast produced by this pattern in thermal images to appear poor or dim [25]. Therefore, through varying materials, surface emissivity and thermal conductivities, calibration targets will have distinctive features in the IR spectrum, allowing for the geometric calibration of IR cameras [26].

## 2. Literature Review

Thermal camera geometric calibration targets are classified into two categories, namely passive and active calibration targets. Passive targets mainly rely on the difference in emissivity values of the pattern, and may employ an external heater to allow the pattern to be visible in the IR spectrum. External heaters could be sunlight, a flood lamp, or an electric fan heater that is independent of the calibration target. Contrarily, active targets rely on both the emissivity difference and the use of a built-in heater to magnify the contrast in a thermal image. This complicates the fabrication of the calibration target, as active targets require electrical work in addition to the selected pattern. Being an active research field, numerous developments have been presented in the literature, with different techniques and calibration targets to geometrically calibrate IR cameras.

One of the most popular approaches involves the typical ink/laser paper-printed square checkerboard pattern used to calibrate visible cameras. However, the pattern is heated using a flood lamp, allowing the pattern to be visible in the IR spectrum [27,28]. A similar study involved the use of a flood lamp to heat the square paper-printed checkerboard target, but here the target was placed on a ceramic tile to retain heating for a longer duration [29]. Similarly, a study geometrically calibrated an IR camera using the checkerboard pattern printed on matt paper and placed on a wall to ensure its flatness, while an IR-emitting lamp was used instead of the typical flood lamp [30]. Another study analyzed the performance of two calibration targets, a paper-printed checkerboard pattern heated using a flood lamp, and a cardboard mask of regularly cut squares placed on a monitor to intensify the contrast of the thermal image [31]. Likewise, a square grid was cut from a black painted K-line material, where a polyethylene foam board fixed between surface glossy papers was used to calibrate an IR camera without any source of heating [32]. Some studies involved the use of a paper-printed checkerboard pattern, along with aluminum foil tape placed on white squares, to provide a greater difference in emissivity and a higher contrast thermal image [33,34]. A few studies implemented the printed checkerboard technique, but not on paper; instead, laser-printed black ink squares on a thin aluminum sheet was implemented [35], or digitally printed thick black ink squares on a thin sandblasted aluminum plate exposed to a halogen lamp or sunlight for heating [25]. A recent study proposed the printing of the checkerboard pattern on a Dibond^®^ panel, an aluminum composite material, but the printing was made using a flatbed ultraviolet (UV) industrial advertisements printer, and heated using an electric fan heater [6].

Apart from printing the square checkerboard pattern, few studies analyzed the effectiveness of two different taped calibration targets, an aluminum plate with high emissivity square tape, and cardboard with low emissivity square aluminum foil tape, with the targets being heated using a monitor display [36,37]. Similarly, a study involved the geometric calibration of thermal cameras, part of a target tracking project, and used a square checkerboard pattern made of a white highly emissive tape placed on an aluminum foil base [38]. Likewise, a recent study tested a square checkerboard pattern built from thick aluminum foil and thick thermocol (polystyrene) square pieces covered with white polyvinyl acetate (PVA) glue [19]. Geometric calibration was performed indoors, without external heating on long-wave infrared (LWIR) and mid-wave infrared (MWIR) cameras. Other recent targets involved painting the square checkerboard pattern on aluminum plates and using Peltier modules, also known as thermoelectric modules, fixed at the back of the calibration rig to either heat and cool the black and white painted squares, respectively [39], or to heat the entire calibration board [40]. Some studies made use of the high thermal conductivity and very low emissivity properties of copper, by milling square checkers on a printed circuit board (PCB) while using a hair dryer to heat the target [41], spraying highly emissive black ink squares on a copper plate and exposing the checkerboard target to sunlight [42]; alternately, polished copper squares were printed on an epoxy plate, and then the target was placed on a heated ceramic tile and an ice block to assess whether heating or cooling the pattern will result in a higher contrast thermal image and more accurate calibration results [43]. One study involved the production of a novel square calibration target by manufacturing a metallic net and placing it on top of a plastic board. With the use of a heat gun, the metallic net appeared brighter in a thermal image over the cooler plastic board [44].

Other than the simple square checker patterns, circular patterns of symmetrical and asymmetrical configurations have been developed in the literature to calibrate thermal cameras. Circular calibration targets are usually complicated in terms of manufacturing and recognition algorithms, where they require specifically tailored codes to recognize the circular pattern and estimate their center points. However, such circular calibration targets tend to deliver better calibration results over square or checker patterns [6], since the algorithm traces the circular patterns to estimate their center points and uses them as control points, rather than detects the corner points, which are not clearly defined in the square checkerboards. Being the most popular circular calibration target approach, burning lamps are aligned symmetrically on a wooden plank [45] or a black-coated plastic board [46]. When switched on, the lamps emit more IR radiation than the base plate, allowing it to be visible to the IR camera. This makes it easier to be recognized by the geometric calibration algorithm. A similar approach was developed [47], but IR-emitting electric resistors were used, fixed and aligned symmetrically on a metallic plate instead of the burning lamps on a wooden or a plastic board. Rather than manufacturing an active calibration target that is electrically operated, passive circular calibration patterns were developed by laser printing an asymmetric black ink circular pattern on a white glossy Dibond^®^ panel and exposing it to sunlight [48], or UV printing a symmetrical circular pattern on a Dibond^®^ plate and heating the plate using an electric fan heater [6] or by exposing it to sunlight [49]. A different approach resided in creating a symmetrical calibration target built purely from aluminum, heated using an electric heater, and divided into two pieces. The two pieces consisted of an anodized perforated circular grid to have a shiny silver appearance, and a black coated base, resulting in different emissivity values being visible to the IR camera [50]. Another method created a target by covering a wooden plank using thin aluminum foil, then perforating a symmetrical circular grid in a black card and placing it on top of the aluminum-covered wooden plank, allowing the aluminum sheet to be only visible from the perforated circular regions [51]. One technique involved creating an asymmetric grid by laser cutting the circular pattern from cardboard [52] or acrylic glass, and heating the target using a heat gun [53].

There are two main sources of error that affect the geometrical calibration of visible and IR cameras, namely the efficacy of the calibration target [54] and the ambiguity in detecting the control points of the images [55]. These issues tend to magnify when dealing with thermal cameras, as thermal images are typically of lower contrast and modest in quality than visible images [25]. With radial and tangential lens distortions affecting the images, imprecise calibration results in further errors that affect the certainty of the geometric calibration study [56,57]. While few studies described the detailed construction of the used geometric calibration targets [6,19,25,37,47,49,51], several studies shared little to no details on how the calibration rigs were constructed. Furthermore, many studies involved the manual construction of the targets, such as the manually cut square grids [31] or the manual aluminum foil and tape placements on the targets [19,33,34,36,38]. Additionally, with the circular patterns already being a more complicated calibration approach than square patterns, due to the need of specifically tailored algorithms, using burning lamps or resistors to construct an electrically operated active calibration board further complicates the procedure. One way to assess the accuracy of the calibration procedure is by computing a metric in pixels, which is usually measured by the adopted calibration toolbox, called the reprojection errors (RE). Table 1 summarizes the different geometric calibration techniques performed on thermal cameras, showing how the calibration targets are constructed, along with the calibration conditions, the emissivity of the targets and the accuracy of the procedure, if reported. The table also shows the optical and thermal images of the geometric calibration targets, if reported in the literature. This active field of research, with many creative developments, all has a clear goal of fabricating a calibration target that provides the fewest reprojection errors possible. It can be seen that the reprojection errors for the square checkerboard pattern range from 0.100 to 1.274 pixels, with the lowest error of 0.100 pixels being reported in [25], while the reprojection errors for the circular pattern reported in the literature range from 0.052 to 0.588 pixels, with the lowest error of 0.052 pixels being reported in [6].

The thorough literature review discussed earlier indicates the importance of having high emissivity contrast within a checkerboard pattern to improve the consistency in detecting or estimating the position of the control points within a planar target. Other than the thorough literature review of the geometric calibration targets that was developed over the years, this study aims at proposing a practical checkerboard calibration target with a precise pattern that is easily constructed through controlled techniques, while providing maximum contrast in emissivity to reliably detect the control points of interest, that is, to attain the lowest RE possible, which makes the proposed target and procedure as practical as possible for different applications requiring geometrical information from thermal cameras. Thus, this research proposes two simple and passive square checkerboard calibration targets, as they lend themselves to commercially available calibration toolboxes. To improve the emissivity contrast within a checkerboard pattern, a polished stainless steel plate was selected as a base plate, while the square checkerboard pattern was laser-engraved and UV-printed on both sides of the plate to form two different calibration targets from one plate. An emissivity check following standard procedures was performed on the targets, in order to ensure that the squares had relatively high emissivity contrast, along with exposing the targets to direct sunlight before capturing the thermal images to guarantee high quality and high contrast thermal images needed for the geometric calibration of IR cameras. The UV-printed checkerboard pattern on a polished metallic alloy offered the lowest mean reprojection error (MRE) of 0.057 pixels and the lowest reprojection root mean square error (RMSE) of 0.063 pixels, with a standard deviation that was lower than 0.003 pixels. It delivered better accuracy than any other checkerboard calibration target, with comparable results to the best prominent circular pattern results reported in the literature.

## 3. Calibration Model and Reprojection Errors

### 3.1. Calibration Model

**Figure 2:**
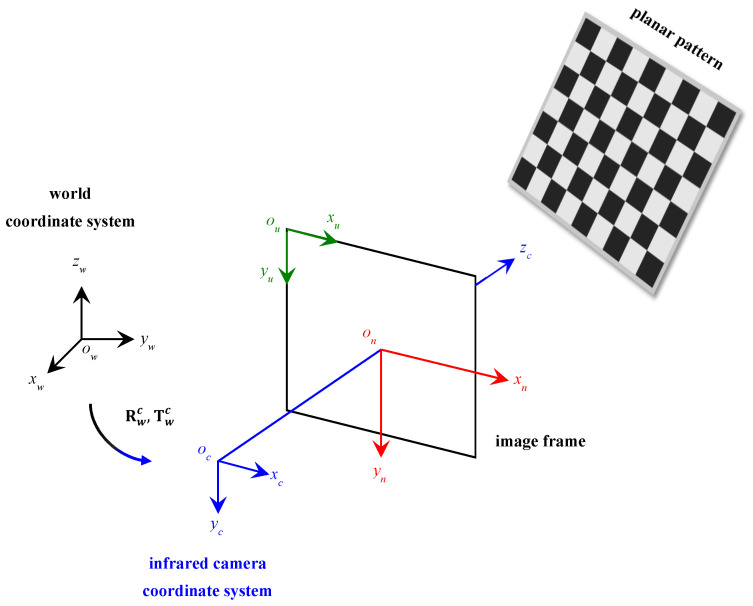
A schematic representation of the camera calibration setup following Zhang’s model [58] by mapping the 3D world scene into an image plane using a randomly oriented planar checkerboard target.

To recover and extract 3D information from a 2D image, camera calibration was required to assess the camera’s parameters in order to transpose data in the 2D image frame to the 3D world coordinate frame. The most popular and robust calibration procedure technique was developed by Zhengyou Zhang [58]. The method relies on imaging a planar pattern of well-known dimensions at a minimum of two different random orientations relative to the camera’s lens, as shown in the calibration schematic representation in Figure 2. The 3D world coordinate reference system is represented by $o_{w}\;x_{w}\;y_{w}\;z_{w}$, the 3D camera coordinate reference system is represented by $o_{c}\;x_{c}\;y_{c}\;z_{c}$, and the 2D image frame of reference is described by $o_{u}\;x_{u}\;y_{u}$. Following the pinhole camera model approximation, a randomly positioned point in 3D world coordinate space projects a 2D point in the image frame, as implemented in [59,60], as follows:

(1) $$s\left[\begin{array}{c} x_{u}\\ y_{u}\\ 1 \end{array}\right]=A\left(I|0\right)H_{w}^{c}\left[\begin{array}{c} x_{w}\\ \begin{array}{c} y_{w}\\ \begin{array}{c} z_{w}\\ 1 \end{array} \end{array} \end{array}\right]$$

where s is an arbitrary scaling coefficient, A is a 3×3 intrinsic matrix representing the camera’s internal parameters projecting a point $p_{c}=\left(x_{c},y_{c},z_{c}\right)$ in the camera’s coordinate frame to a point $p_{u}=\left(x_{u},y_{u}\right)$ in the 2D image frame, and $H_{w}^{c}$ is a 4×4 extrinsic matrix consisting of an orthogonal rotational matrix and a translational vector linking the world coordinate system to the camera’s coordinate system. Finally, (I|0) is a 3×4 augmented transformational matrix used to transform points in the 3D camera coordinate frame to the 2D image frame, where I is a 3×3 identity matrix and 0 is a 3×1 zero matrix. One way to simplify Equation (1) is by splitting it into two equations. The first equation represents the extrinsic parameters of the camera, where it transforms a point in a 3D world coordinate frame to the 3D camera coordinate frame of reference, as follows:

(2) $$\left[\begin{array}{c} x_{c}\\ \begin{array}{c} y_{c}\\ \begin{array}{c} z_{c}\\ 1 \end{array} \end{array} \end{array}\right]=\left[\begin{array}{cc} R_{w}^{c} & T_{w}^{c}\\ 0^{T} & 1 \end{array}\right]\left[\begin{array}{c} x_{w}\\ \begin{array}{c} y_{w}\\ z_{w} \end{array}\\ 1 \end{array}\right]$$

where $R_{w}^{c}$ is the 3×3 orthogonal rotational matrix, $T_{w}^{c}$ is the 3×1 translational column vector, while $0^{T}$ is a 1×3 zero row vector used to transform the 3×4 matrix into a 4×4 extrinsic matrix $H_{w}^{c}$ for uniform multiplication in a homogeneous coordinate system, as shown below:

(3) $$H_{w}^{c}=\left[\begin{array}{cc} R_{w}^{c} & T_{w}^{c}\\ 0^{T} & 1 \end{array}\right]=\left[\begin{array}{cc} \begin{array}{cc} \begin{array}{cc} \begin{array}{c} \begin{array}{c} \begin{array}{c} r_{11}\\ r_{21} \end{array}\\ r_{31} \end{array}\\ 0 \end{array} & \begin{array}{c} \begin{array}{c} \begin{array}{c} r_{12}\\ r_{22} \end{array}\\ r_{32} \end{array}\\ 0 \end{array} \end{array} & \begin{array}{c} \begin{array}{c} \begin{array}{c} r_{13}\\ r_{23} \end{array}\\ r_{33} \end{array}\\ 0 \end{array} \end{array} & \begin{array}{c} \begin{array}{c} \begin{array}{c} t_{x}\\ t_{y} \end{array}\\ t_{z} \end{array}\\ 1 \end{array} \end{array}\right]$$

The second equation involves the projection of points from the 3D camera coordinate frame to the 2D undistorted image pixel frame. It involves the intrinsic parameters of the camera, as follows:

(4) $$s\left[\begin{array}{c} x_{u}\\ y_{u}\\ 1 \end{array}\right]=A\left[\begin{array}{c} x_{c}\\ y_{c}\\ z_{c} \end{array}\right]$$

where

(5) $$A=\left[\begin{array}{ccc} \alpha_{x} & c & u_{0}\\ 0 & \alpha_{y} & v_{0}\\ 0 & 0 & 1 \end{array}\right],$$

$\alpha_{x}$ and $\alpha_{y}$ are the scaling factors function in the x and y axes of the camera’s focal length in pixels of the image, respectively; $u_{0}$ and $v_{0}$ are the principal point or the optical center in pixels of the image, and c is the skew coefficient measuring the skewness of the image frame axes. It should be noted that the skew coefficient c is zero when the image axes are perpendicular, which is the case in this model, as presented in Figure 2.

The camera matrices specified above do not account for distortions caused by the curvature of the lens, as the ideal pinhole model does not have a lens. To accurately calculate the projection, and thus correctly model the camera, the calibration model must account for radial and tangential lens distortions. Radial distortions arise when the light rays or IR radiation bend near the edges more than they bend at the camera’s optical center, while tangential distortions arise when the camera’s sensor detector or simple image plane is not parallel to the lens [61]. Let (x,y) be the ideal distortion-free normalized image frame coordinates, and $\left(\overset{ˇ}{x},\overset{ˇ}{y}\right)$ be the distorted normalized image frame coordinates. The lens distortion, radial and tangential, may be modeled using the following polynomial equation [61]:

(6) $$\overset{ˇ}{x}=x+\underset{\mathrm{Radial}}{\underset{︸}{x\left(k_{1}r^{2}+k_{2}r^{4}+k_{3}r^{6}\right)}}+\underset{\mathrm{Tangential}}{\underset{︸}{2p_{1}xy+p_{2}\left(r^{2}+2x^{2}\right)}}$$

and

(7) $$\overset{ˇ}{y}=y+\underset{\mathrm{Radial}}{\underset{︸}{y\left(k_{1}r^{2}+k_{2}r^{4}+k_{3}r^{6}\right)}}+\underset{\mathrm{Tangential}}{\underset{︸}{2p_{2}xy+p_{1}\left(r^{2}+2y^{2}\right)}}$$

where $r^{2}=x^{2}+y^{2}$, $\left(k_{1},k_{2},k_{3}\right)$ are the radial distortion coefficients, and $\left(p_{1},p_{2}\right)$ are the tangential distortions coefficients of the lens.

### 3.2. Reprojection Errors

The metric used to assess the accuracy of camera calibration is the reprojection error. Reprojection errors are distances measured in pixels between the detected control points, or features extracted from the image with the corresponding reprojected control points, or features of the calibration checker pattern. The reprojected control points in the world coordinates of the calibration pattern could be the corners of squares, centers of squares, or the centers of circles, depending on the calibration pattern used. Through the use of the computed camera parameters, reprojection of the world coordinates control points to image coordinates is possible through a transformation procedure.

Reprojection errors are of two types, the MRE and RMSE of reprojection, which are defined as follows:

(8) $$\mathrm{MRE}=\frac{\sum_{i=1}^{I}\sum_{j=1}^{J}\|p_{ij}-p_{ij}^{\prime }\|}{IJ}$$

(9) $$\mathrm{RMSE}=\sqrt{\frac{\sum_{i=1}^{I}\sum_{j=1}^{J}\|p_{ij}-p_{ij}^{\prime }\|{}^{2}}{IJ}}$$

where $p_{ij}$ is the image point and $p_{ij}^{\prime }$ is the reprojected point through the calibration model. MRE is the average of the Euclidian distance between the detected control points of the image and the reprojected points from the pattern. The RMSE, however, gives an indication of the level of deviation from the detected control points of the image and the reprojected points from the pattern [39]. In the IR camera geometric calibration reported in the literature summarized and listed in Table 1, the MRE and RMSE are interchanged; some studies reported their MRE values, while others reported their RMSE of reprojection values. While both metrics have been used over the years to assess the performance and model errors on different applications, there is no consensus on which criteria to better model the reprojection errors [62]. Some studies argue that the commonly employed sums-of-squares-based errors, such as the RMSE, are often misleading criteria indicators for the average error, making the RMSE an inappropriate, ambiguous and misinterpreted measure of the mean error [63,64]. However, a study argues that the RMSE is not vague in its meaning, and is more appropriate as an indicator for model performance than the mean absolute error (MAE), represented by the MRE in this study, when the error distribution is expected to follow a Gaussian trend [62]. Therefore, without leaning towards a certain evaluation criterion or indicator, a combination of metrics often prove practical in assessing model performance [62]. In order to remain conservative, while also being fair in comparison to the available literature, this research computed both the MRE and the RMSE of all trials, for both the laser-engraved and UV-printed calibration targets, using Equations (8) and (9). Thus, this study did not rely on just a single indicator for the accuracy of the calibration, but offered a verification of the obtained errors by computing the two indicators most widely used for assessing the accuracy of the geometric calibration process.

## 4. Experiment

### 4.1. Calibration Targets Design

As mentioned above in Section 2, the quality, precision and efficacy of the calibration target contribute massively to the accuracy of the geometric calibration performed on the thermographic camera. Additionally, the calibration model discussed in Section 3.1 and implemented in this study, relies on detecting the corners of a square checkerboard pattern, requiring precise alignment and seamless corners between the edges of the squares. Manually constructed checker patterns provide low geometric calibration accuracy, as the squares are not precisely aligned, resulting in high errors in control points recognition. Furthermore, active symmetrical and asymmetrical circular calibration targets are complicated in terms of construction and recognition, in the majority of the cases, as they mandate specially tailored algorithms that are not as user-friendly as the square checkerboard commercially available software toolboxes. The thorough literature review has been leveraged to deliver a practical checkerboard target, offering high geometric calibration accuracy that competes with that of circular calibration targets. Other than its competing accuracy, it offers a practical solution to less experienced end users to apply the IR camera geometric calibration with ease via commercially available software toolboxes tailored for square checkerboard patterns. Thus, the proposed calibration checkerboard targets should be well designed and manufactured to a perfectly flat and polished metallic alloy to offer low surface emissivity prior to adding the pattern. The added (engraved or printed) checkerboard squares should be of a relatively high emissivity value, and perfectly aligned with sharp and clear corners for better defined controlled points. This offers a sufficient emissivity contrast between the squares with well-defined corners in an IR camera field of view.

**Figure 3:**
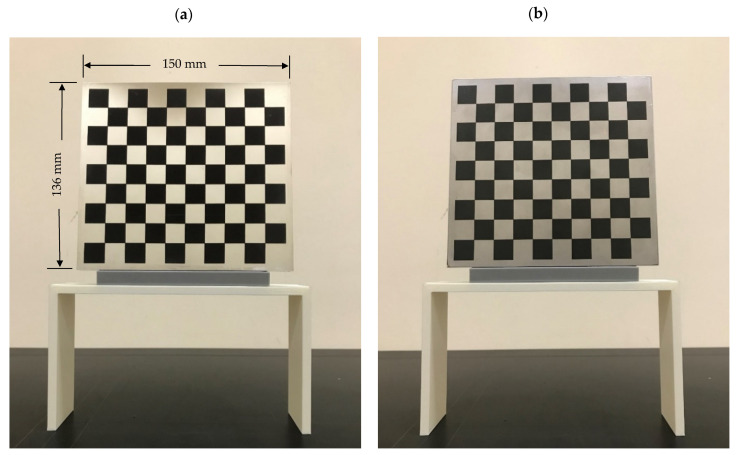
Optical images of (**a**) the laser-engraved and (**b**) the UV-printed checkerboard patterns.

**Figure 4:**
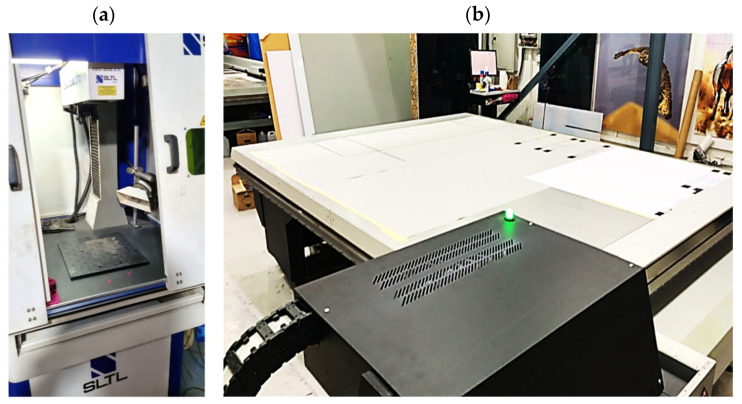
Checkerboard targets fabricated using (**a**) SLTL’s Akshar Fiber Pro Ultra laser engraving machine and (**b**) Canon’s Arizona 1380 XT flatbed UV printer.

Accordingly, this research proposed the use of a polished stainless steel alloy (SS304) flat plate as the base material, with the square checker pattern laser-engraved on one side of the plate and UV-printed on the other side, as shown in Figure 3a,b, respectively. SS304 was selected due to its appealing physical and thermal properties, as detailed in a technical datasheet [65] and listed in Table 2, along with its wide availability in the market at a relatively low cost. It is also an extremely corrosion-resistant alloy, which allows for the preservation of the pattern and its corners over time. A 150 mm×136 mm×6 mm plate was used, and a checker pattern of 10×9 squares having a square size of 14 mm each was selected, leaving a 5 mm space margin between the pattern and the edges of the SS304 plate. The distinctive features or control points of both targets are the corners of the squares, while the difference in the number of rows (even) and columns (odd) is a distinctive feature that permits orientation detection by the calibration toolbox. The laser-engraved pattern was performed using SLTL’s Akshar Fiber Pro Ultra laser engraving machine, shown in Figure 4a. A mirror polishing machine was used to ensured that the plate had a mirror finish and was flat, while the laser engraving machine operated on a maximum plate size of 150 mm×150 mm, and enforced an engraving of 0.3 mm in depth, leaving a brownish-black rough textured engraved pattern, as shown in Figure 3a. The printed pattern, shown in Figure 3b, was performed using Canon’s Arizona 1380 XT flatbed UV printer with a flatbed printer that used UV-curable inks, providing high-value graphic art and industrial printing. The printer, shown in Figure 4b, provides a printing resolution of 1440 dpi (dots per inch) or 56.7 dots per millimeter, delivering a highly accurate square checker calibration pattern with its state-of-the-art sharpness and resolution. It should be noted that the surface of the plate where the pattern was UV-printed was sanded using emery papers of different grades, in order to ensure a smooth surface without any scratches. Both the laser engraving machine and the UV printer required the plate to be flat, which was ensured using the 6 mm thick SS304 plate, as any slight bends or warpage to the plate leads to defective calibration results [66].

### 4.2. Experimental Setup

**Figure 5:**
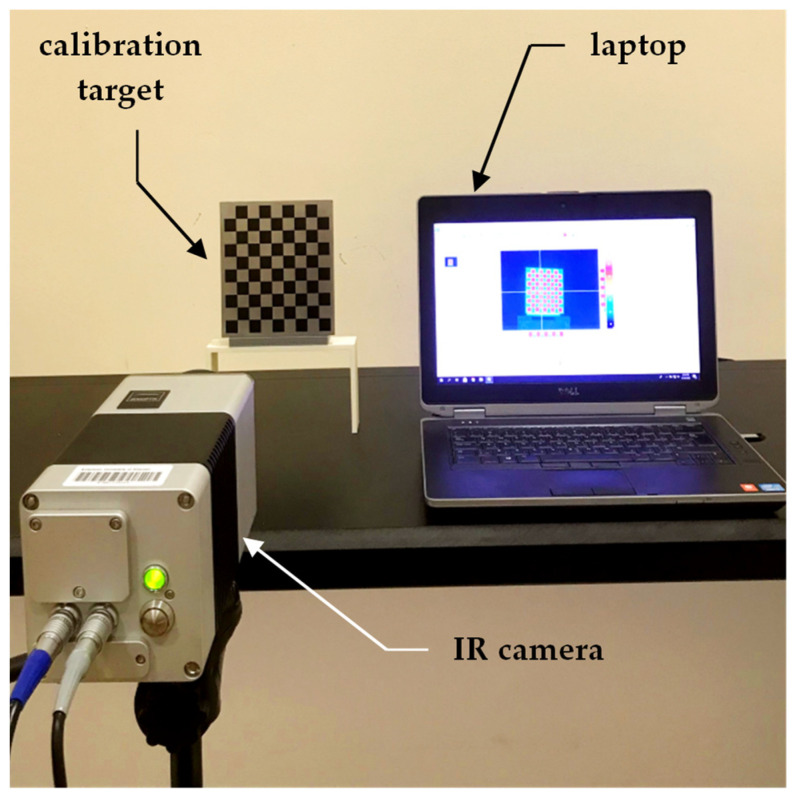
A presentation of the experimental setup used in this study.

The experimental setup, equipped in this study and shown in Figure 5, consisted of placing the calibration targets on a plastic holder, a thermographic camera, and a laptop to control the acquisition of the thermal images. The IR camera model used was the VarioCAM HD Head-600 manufactured by InfraTec, having a resolution of 640×480 pixels. The thermal detector used was an uncooled micro-bolometer focal plane array (UFPA), operating in a photoconductivity system, where the sensor’s material alters its electrical resistance when heated by incident IR radiation of a certain wavelength [67]. Uncooled cameras referred to the uncooled detectors of the thermal camera, where the sensors operated in ambient conditions that required no cooling to cryogenic temperatures, as needed by the cooled quantum detector type. Uncooled IR cameras are usually less expensive, less complicated, and lower in weight compared to cooled IR cameras, which allowed for the introduction of thermal imaging to the mass market [67]. The thermographic camera used in this study was of the LWIR type, and its technical specifications are listed in Table 3.

### 4.3. Emissivity Coefficient

Accurately detecting the positions of the control points in thermal images of the calibration targets relied heavily on the ability to obtain high-quality thermal images. High-quality thermal images depend on the precision of the square checker pattern of the calibration target. For objects to be visible and distinct in a thermal image, the infrared radiation collected by the IR camera from the objects in the scene should be different [6]. The IR signature of an object depends on its temperature distribution and the emissivity of its material [68]. The emissivity coefficient (ε) of a material is a dimensionless coefficient ranging from 0 to 1, which indicates the object’s ability to emit IR radiation in comparison to a blackbody at the same temperature. A blackbody is a theoretical material with ε=1, meaning that it absorbs and perfectly re-emits all incident IR radiation [69]. Therefore, an object with an emissivity value close to zero emits negligible thermal radiation in comparison to an object with an emissivity value close to unity. The emissivity of an object depends on many parameters; these include the material, structure and texture of an object’s surface, the surface temperature of the object, and the wavelength of the thermal radiation collected by the IR camera [70,71]. Thus, the emissivity coefficient is the ratio of the thermal radiation emitted by the imaged object M(T) to the thermal radiation emitted by the blackbody $M_{0}\left(T\right)$ under identical thermal conditions [26], as follows:

(10) $$\varepsilon =\frac{M\left(T\right)}{M_{0}\left(T\right)}$$

It is critical to note that the energy striking opaque surfaces is reflected and/or absorbed. Moreover, one particular case is where the emissivity of the surface does not influence the contactless measurement of the surface temperature, as emitted or reflected IR radiation becomes independent of the object’s surface emissivity. This is when the environmental/background temperature is identical to the object’s temperature [6], causing the target to be indistinguishable in a thermal image. The solution to this issue is to heat the object or place it in an environment that has a different background temperature. For calibration targets, this is usually performed in two ways, by either actively heating the calibration target using electrically powered heaters and performing the calibration imaging indoors, or by acquiring the thermal images outdoors where the sun’s or sky’s radiation reflects off the target and eliminates the need for external heating. This research proposed the acquisition of the calibration target images in an indoor controlled environment after placing the target under direct sunlight for a few minutes, excluding the need for any external heating devices, as well as avoiding any degradation in image quality caused by the reflection of IR radiation when performing outdoor calibration.

The American Society for Testing and Materials (ASTM E1933-14) defines two standard test methods for determining the emissivity of objects when measuring their surface temperatures with IR imaging radiometers [72]. The first method is called the contact thermometer method (CTM). It involves the use of a contact thermometer to measure the temperature of a point or area where the emissivity is to be defined. The emissivity on the infrared imaging radiometer or IR camera’s computer focused on the same spot is adjusted until both readings match. The second technique, called the noncontact thermometer method (NTM), employs a surface-modifying material (SMM) of known emissivity that adheres to the target’s surface. The IR camera’s emissivity is set to the SMM emissivity value, and the temperature value is recorded. Then, the IR camera is focused on a spot on the target adjacent to the SMM where emissivity is to be defined, and the IR camera’s emissivity is adjusted until the temperature reading of this spot matches the one recorded on the SMM. Both methods require a minimum of three repetitions, and the material’s emissivity is the average of these repetitions [72]. Several studies listed in Table 1 reported the emissivity of the calibration targets used. However, only a few studies discussed the method used to obtain the emissivity of the patterns. A recent study adopted the NTM to evaluate the emissivity of a square checker pattern printed on an aluminum composite material, with the SMM being the easily attainable electrical tape [6]. Apart from following the standard NTM while using commercially available electrical tape as the SMM, not knowing the tape’s exact emissivity may introduce errors in the emissivity check.

**Figure 6:**
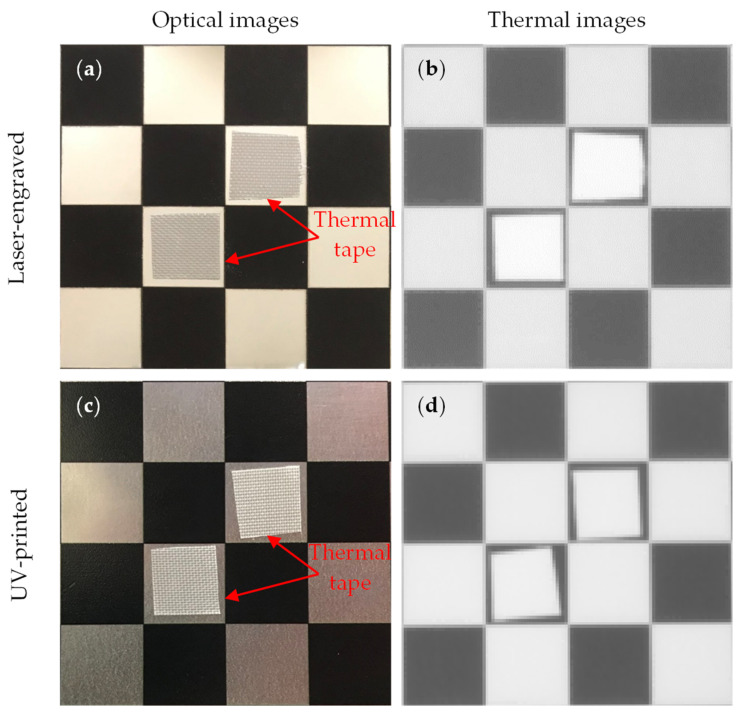
Laser-engraved pattern (**a**) optical and (**b**) thermal images and UV-printed pattern (**c**) optical and (**d**) thermal images.

This research followed the NTM approach to estimate the emissivity of the laser-engraved and UV-printed checker patterns, using thermal emission tape as the SMM. The thermal emission tape, manufactured by Testo SE & Co., has an emissivity of ε=0.95. Two locations were selected to measure the emissivity of the patterns for both targets, as shown in Figure 6a,b for the laser-engraved target and Figure 6c,d for the UV-printed target, in order to assure certainty. After adhering the SMM, the targets were placed under direct sunlight for a few minutes for heating, then thermal imaging was performed indoors. The emissivity check was repeated four times, meeting the requirement set by the ASTM E1933-14 standard, with the average emissivity values of the patterns reported in Table 4. It was seen that UV printing the pattern resulted in a greater difference in emissivity between the base metal plate and the pattern, having $\varepsilon_{min}=0.239$ and $\varepsilon_{max}=0.931$, while laser engraving the pattern resulted in $\varepsilon_{min}=0.172$ and $\varepsilon_{max}=0.811$, respectively. The emissivity of the bare metal on the laser-engraved target was lower than that on the UV-printed one, as the earlier was polished by the supplier to attain a mirror surface finish, while the latter was only sanded using different emery paper grades to ensure a smooth surface. It should be noted that the proposed UV-printed checkerboard pattern on the stainless steel plate showed a greater difference in emissivity compared to a similarly UV-printed pattern on an aluminum composite board in a previous study, where $\varepsilon_{min}=0.67$ and $\varepsilon_{max}=0.83$ [6]. This improved difference in emissivity was mainly due to the selection of SS304 as a base plate and polishing its surface to obtain a mirror-like finish, resulting in a much lower $\varepsilon_{min}$ compared to the previously mentioned study. Furthermore, this improvement in emissivity difference resulted in higher quality thermal images, as the printed pattern was clearly distinguishable from the base material, with higher precision in the recognition of the corner control points.

### 4.4. Experimental Procedure

As mentioned in Section 4.3, to introduce high contrast between the calibration pattern and the background environment in indoor thermal imaging, the targets were exposed to direct sunlight for a few minutes, allowing the patterns to heat up. When observed by the IR camera, the temperature of the targets was in the range of 45–50 °C. It should be noted that the temperature of the targets when left under sunlight was not controlled, but was left for enough time to display good contrast in the thermal image for clear corner detection; the earlier mentioned range was just an observation made for the records. The targets were then brought to an indoor environment where the temperature was around 22 °C, placed on a plastic holder, and imaged using the thermal camera. To ensure accurate calibration results, multiple images of the calibration checkerboards at different 3D orientations were needed. Thus, by moving the calibration target holder in different positions and rotating the camera in different orientations, the thermal images of the checkerboard target were obtained. Due to the difference in temperature between the calibration checkerboard and the indoor background environment, the calibration targets cooled down in a few minutes, degrading the quality of the captured thermal images and their sharpness. Thus, the solution to this issue was to acquire 6 images at a time, which took around 5 min to adjust for the random orientations, followed by the process of placing the targets under sunlight and then acquiring the other 6 images 5 times until 30 thermal images of the calibration target at different orientations were obtained. This procedure was repeated 9 times for each checkerboard target.

**Figure 7:**
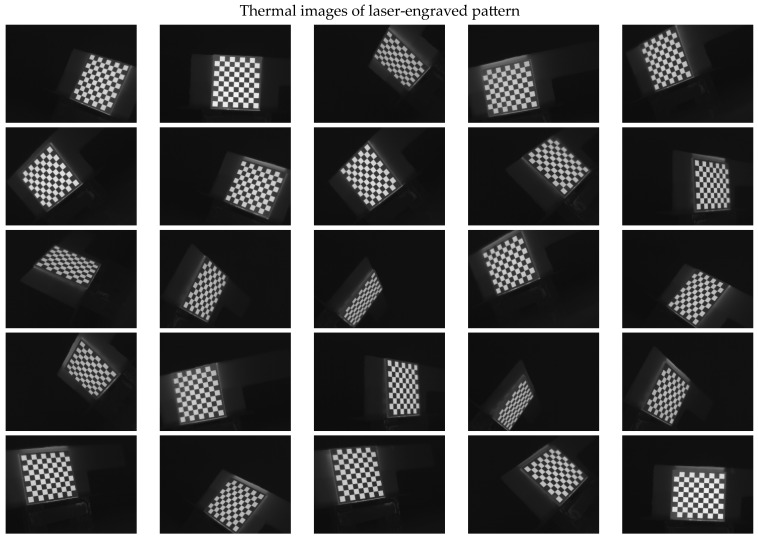
Thermal images of the laser-engraved checkerboard pattern viewed at random orientations for the IR camera geometric calibration.

**Figure 8:**
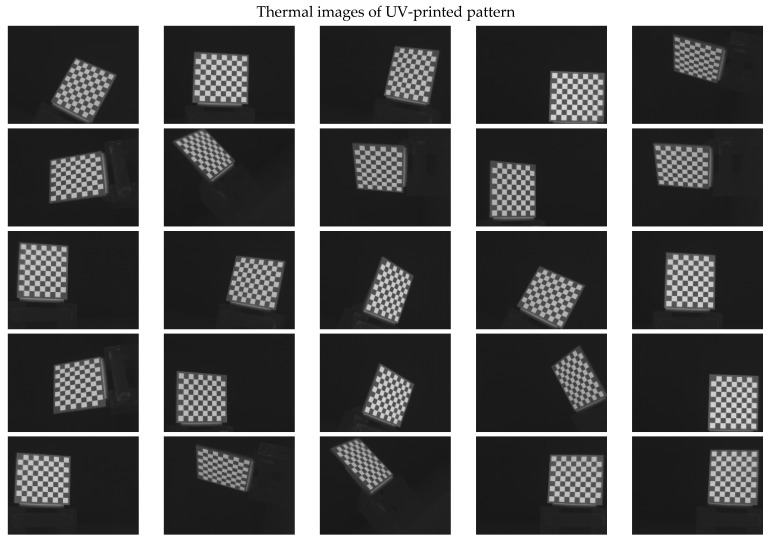
Thermal images of the UV-printed checkerboard pattern viewed at random orientations for the IR camera geometric calibration.

The 30 thermal images, considered as 1 trial per checkerboard target, provided the thermal map as temperature readings for the entire FOV of the thermal camera lens. These thermal images were then converted to grayscale images and saved in the Tag Image File Format (TIFF), due to the format’s high quality and universal compatibility. The 30 images were then loaded into the single-camera calibrator toolbox using MATLAB [73], where the standard single-camera model was selected, the square size was specified as 14 mm, and the camera was calibrated for radial and tangential distortions. To improve the calibration results, it was recommended to remove the images that were blurry or showed high errors [73], and to repeat the calibration. Therefore, 5 images showing the highest errors were excluded from the 30 images, leaving 25 images per trial for the laser-engraved and UV-printed checkerboards, as shown in Figure 7 and Figure 8, respectively. This calibration process was repeated 9 times for both the laser-engraved and UV-printed patterns. It should be noted that at least 3 checkerboard calibration target images were required, but 10 to 20 thermal images of the target were captured, while the lowest performing images were eliminated to obtain the best checkerboard calibration results [73]. However, as more captured images would not harm but affirm the calibration results, 30 thermal images of the target were captured per trial, and the 5 images with the lowest calibration results were excluded.

## 5. Results and Discussion

**Figure 9:**
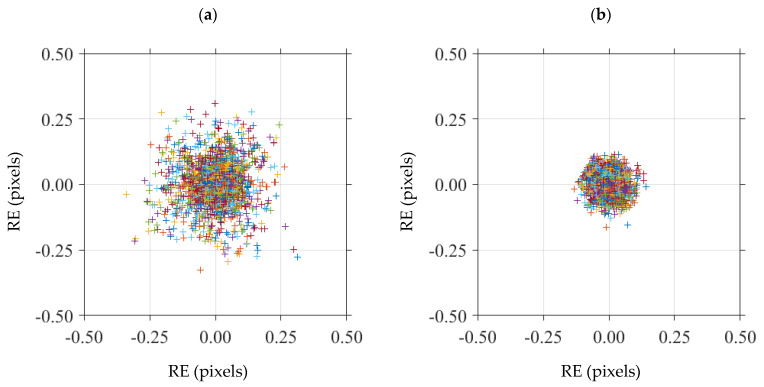
Distribution of the reprojection errors (RE) of all detected image points, using one calibration trial per selected checkerboards. (**a**) Laser-engraved checkerboard; (**b**) UV-printed checkerboard.

To assess and compare the performance of the calibration targets, the internal parameters of the IR camera were evaluated. The calibration targets were observed at different orientations, and then the images were fed to the calibration toolbox for feature extraction, identification of control points, computing the intrinsic and extrinsic camera parameters, and finally for evaluating the reprojection errors to assess the accuracy of the calibration target. Figure 9a,b present RE values obtained between the detected and reprojected corners of the squares for one of the trials on the laser-engraved and UV-printed checkerboard targets, respectively. It was seen that the reprojection errors obtained by the laser-engraved pattern were more dispersed, with significant outliers over the more consistent and clustered reprojection errors obtained from the UV-printed pattern. This indicates that the UV-printed pattern was more precise, with clearer square corners than those of the laser-engraved pattern, resulting in a more accurate geometric calibration process.

**Figure 10:**
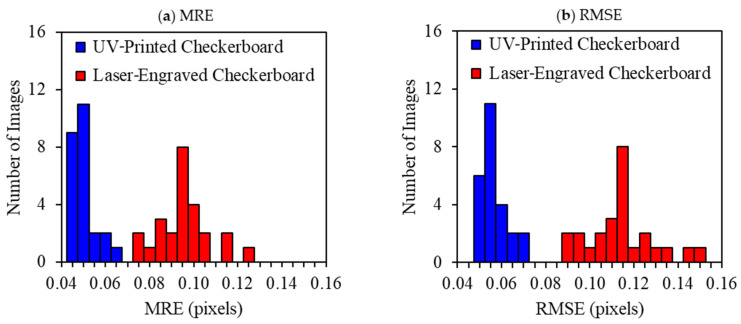
Histogram of (**a**) mean reprojection errors (MRE) and (**b**) root mean square errors (RMSE) of reprojection, using thermal images of calibration trial number 9 per selected checkerboards.

In the visible camera calibration for computer vision applications, obtaining reprojection errors that are less than one pixel is considered suitable and widely acceptable [73]; only in exceedingly critical applications, reprojection errors that are less than 0.1 pixels are required [6]. Table 5 summarizes the reprojection errors, the MRE and RMSE, attained for the nine trials performed on each calibration target, along with their averages and standard deviations. Furthermore, a histogram of the calibration reprojection errors computed by one of the trials of each calibration target was plotted and shown in Figure 10. It was noticed that the MRE, represented by Figure 10a, and the RMSE, represented by Figure 10b, obtained by the UV-printed checker target was less than half of that computed by the laser-engraved target. This indicates that UV printing provided a more precise pattern over laser engraving.

Table 1 summarizes the available literature on this topic, where it also lists the reprojection errors computed by a few of the studies. It can be seen that the reprojection errors for the square checkerboard type range from 0.100 to 1.274 pixels, with the lowest reprojection error, as MRE, of 0.100 pixels attained by digitally printing a thick black ink square checker pattern on a sandblasted aluminum plate and performing the calibration outdoors by exposing the plate to direct sunlight [25]. Moreover, the circular calibration targets produced reprojection errors ranging from 0.052 to 0.588 pixels, displaying a more accurate but more complicated calibration technique over the square pattern method, due to its confined range and lower computed reprojection errors. The lowest reprojection error, an RMSE of 0.052 pixels, was attained using a circular pattern that was UV-printed on an aluminum composite plate, with the images acquired indoors after heating the target with an electrical fan [6]. This study proposed two square checkerboard targets, namely a laser-engraved pattern and a UV-printed pattern, on a polished stainless-steel alloy, then exposing the boards to direct sunlight for heating before capturing the thermal images indoors. From Table 5, it can be seen that the laser-engraved checker target produced an MRE in the range of 0.098–0.137 pixels and an RMSE in the range of 0.117–0.164 pixels over nine different calibration repetitions, with an average MRE of 0.119 pixels with a standard deviation of 0.013 pixels, and an average RMSE of 0.142 pixels with a standard deviation of 0.016 pixels. Similarly, the UV-printed target produced MRE values in the range of 0.053–0.059 pixels and RMSE values in the range of 0.059–0.066 pixels over the nine different calibration repetitions, with an average MRE of 0.057 pixels and a standard deviation of less than 0.003 pixels and an average RMSE of 0.063 pixels with a standard deviation of less than 0.003 pixels. This shows that the UV-printed target is more precise than the laser-engraved target, with lower reprojection errors and one order of magnitude lower standard deviations. With the experimental conditions and procedure all being fixed, such as the target material, checkerboard pattern, thermal camera along with the heating source, indoor conditions and number of thermal images captured per target, the superior calibration accuracy of the UV-printed target is mainly attributed to the precision of the printed square corners to be detected and extracted by the calibrator toolbox, where Figure 6c displays sharper corners over Figure 6a, along with improved emissivity differences between the polished base surface and the printed pattern. Nonetheless, the proposed calibration rigs, whether laser-engraved or UV-printed, provide better and more accurate results in terms of reprojection errors, when comparing them to the available literature that employed the square checker calibration patterns. Additionally, the UV-printed checker pattern produced better results than most circular grid calibration techniques, while producing almost similar results to the lowest reprojection error computed by a circular grid pattern. This proves that the less complicated well-known square checkerboard pattern with improved emissivity contrast delivers desirable reprojection error values. Finally, by repeating the calibration procedure nine times, an average MRE of 0.057 pixels along with an average RMSE of 0.063 pixels, with a standard deviation of less than 0.003 pixels in both was achieved, indicating the consistency of the UV-printed checkerboard target.

## 6. Conclusions

A thorough review of IR camera geometric calibration targets available in the literature, along with their respective results, was summarized and leveraged to deliver a recommended practice for establishing precision square checkerboard calibration targets with relatively high emissivity contrast. The main challenge to overcome was having a precise calibration target with well-defined control points that were clearly visible in the IR spectrum. The square checkerboard target should have a high emissivity contrast, along with clearly defined corners. Thus, this research presented two passive checkerboard targets used to accurately calibrate IR thermal cameras. A square checker pattern was laser-engraved on one side of a polished stainless-steel plate, while the same pattern was UV-printed on the plate’s opposing surface. A thick stainless steel alloy plate was used as the base material to ensure a flat calibration target while displaying a substantial difference in emissivity between its surface and the checker patterns. The targets were placed under direct sunlight for a few minutes, and then imaged using the IR camera in an indoor environment. Calibration was performed to eliminate radial and tangential distortions caused by the thermal camera’s lens, while the accuracy of the procedure was assessed on the basis of computed reprojection errors and compared to the available literature on this topic. The experimental procedure was repeated nine times on each calibration target, and the results indicated accuracy and consistency for both patterns. Average MREs of 0.119 and 0.057 pixels, along with standard deviations of 0.013 and 0.003 pixels, were attained for the laser-engraved and UV-printed patterns, respectively. Similarly, average RMSEs of 0.142 and 0.063 pixels, along with a standard deviation of 0.003 pixels, were attained for the laser-engraved and UV-printed patterns, respectively. This shows that the proposed calibration rigs, whether laser-engraved or UV-printed, provide better and more accurate results in terms of reprojection errors, when compared to those in the available literature using the square checker calibration patterns. Additionally, the UV-printed checker pattern produced better results than most circular grid calibration techniques, while producing almost comparable results to the lowest reprojection error computed by a circular grid pattern. This establishes that the practical square checkerboard pattern with improved emissivity contrast delivers desirable MRE/RMSE values, making it an attractive solution for less experienced end users, without the need to use a circular pattern and their tailored recognition algorithms, and relying only on commercially available tools for the geometric calibration of IR cameras using conventional square checker targets.

## Figures and Tables

**Table 1:** Summary of reported thermal camera geometric calibration targets. Pattern: S (square) and C (circular); condition: I (indoor) and O (outdoor); ε_min_/ε_max_: (lowest/highest emissivity coefficients of pattern); heating: N/A (not available) and NI (no information); RE (reprojection error); RMSE (root mean square error of reprojection); MRE (mean reprojection error).

Pattern	Make	Condition—Heating	ε_min_/ε_max_	RE [Pixels]	Optical Image	Thermal Image
[27]—S	Paper-printed checkerboard pattern	I—Flood lamp	–	–	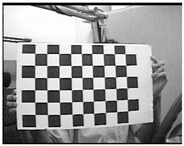	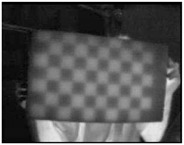
[28]—S	Paper-printed checkerboard pattern	I—Flood lamp	–	–	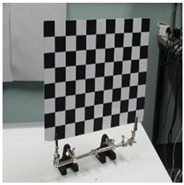	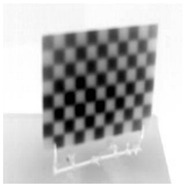
[29]—S	Paper-printed checkerboard pattern taped on a ceramic tile	I—250 W Flood lamp	–	RMSE: 0.410–0.480	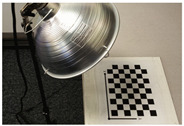	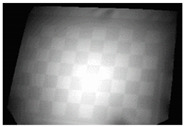
[30]—S	Checkerboard pattern printed on matt paper and taped to a wall	I—IR emitting lamp	–	RMSE: 0.600–0.714	–	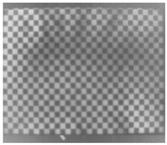
[31]—S	Paper-printed checkerboard pattern	I—500 W Flood lamp	–	MRE: 0.804–1.274	–	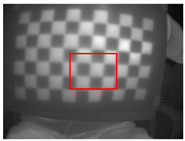
	Cardboard mask of regularly cut squares	I—Monitor display	–	MRE: 0.284–0.324	–	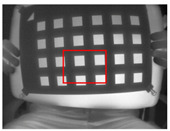
[32]—S	Black K-Line board of regularly cut squares	I—N/A	–	–	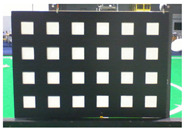	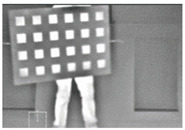
[33]—S	Paper-printed checkerboard pattern with aluminum foil taped on the white squares	I—N/A	–	–	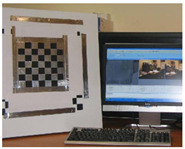	–
[34]—S	Paper-printed checkerboard pattern with aluminum foil taped on the white squares	I—Radiator	–	–	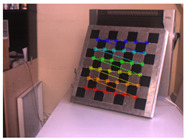	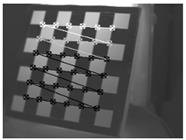
[35]—S	Laser-printed checkerboard pattern on a thin aluminum sheet	I—NI	–	–	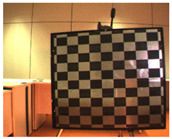	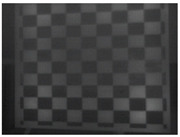
[25]—S	Digitally printed checkerboard pattern on a thin sandblasted aluminum plate	I—Halogen lamp O—Sunlight	0.21/-	RMSE: 0.200–0.370 RMSE: 0.100–0.220	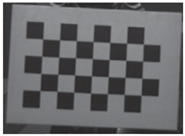	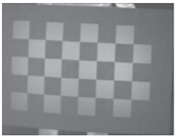
[6]—S	UV-printed checkerboard pattern on a Dibond (aluminum composite) panel	I—Electric fan heater	0.67/0.83	RMSE: 0.233	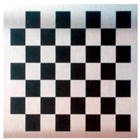	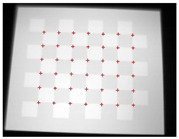
[36]—S	High emissivity squares tape placed on a thin aluminum plate Low emissivity aluminum foil squares tape placed on cardboard	I—N/A I—Monitor display O—Clear cold sky	–	–	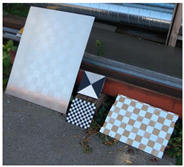	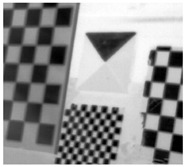
[37]—S	High emissivity insulating squares tape placed on a polished aluminum plate	I—External heater	–	–	–	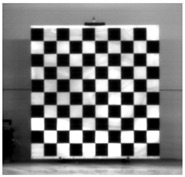
	Low emissivity aluminum foil squares tape placed on a cardboard	I—Monitor display	–	–	–	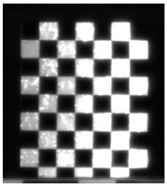
[38]—S	High emissivity squares tape placed on an aluminum foil base	I—NI	–	–	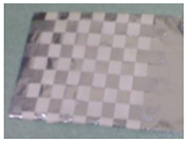	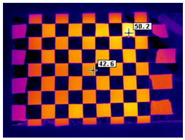
[19]—S	Precisely cut thick thermocol squares coated with white glue along with thick aluminum foil squares adhered to a thick thermocol board	I—N/A	0.03/0.99	MRE: 0.310–0.400	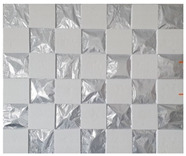	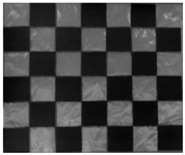
[39]—S	Painted black and white aluminum alloy squares forming a checkerboard pattern and fixed to a wall	I—Peltier module	–	MRE: 0.230	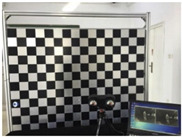	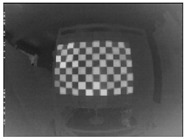
[40]—S	Painted black and white aluminum alloy squares forming a checkerboard pattern and fixed to a wall	I—Peltier module	–	RMSE: 0.280	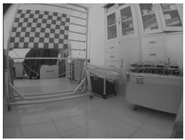	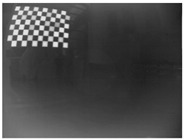
[41]—S	Milled copper squares on a printed circuit board (PCB) base	I—Hair dryer	–	–	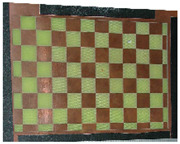	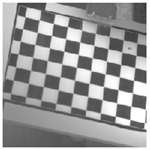
[42]—S	High emissivity black squares painted on a low emissivity copper base plate	O—Sunlight	0.09/0.98	MRE: 0.480–0.650	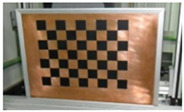	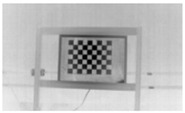
	Low emissivity copper squares on a high emissivity black base plate	O—Sunlight	0.09/0.98	MRE: 0.330–0.450	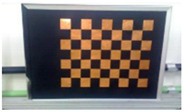	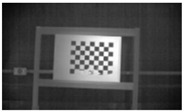
[43]—S	Printed polished copper checkerboard pattern on an epoxy plate	I—N/A I—Hot ceramic plate I—Ice block	–	–	–	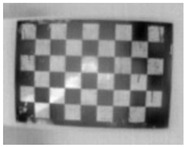
[44]—S	Metal square net placed on a plastic board	I—Heat gun	–	MRE: 0.175–0.177	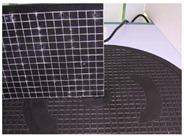	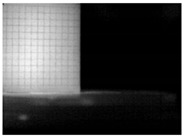
[45]—C	Circular burning lamps fixed on a wooden plank	I—Burning lamps	–	–	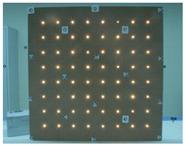	–
[46]—C	Miniature bulbs attached to a black plastic board	I—Light bulbs	–	MRE: 0.588	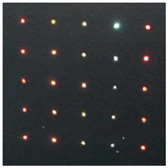	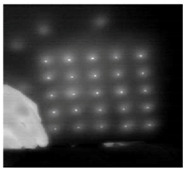
[47]—C	Printed checkerboard pattern mounted on a metallic plate with IR-emitting resistors attached at the centers of the squares, forming a circular calibration target	I—Electric resistorsO—Electric resistors	–	–	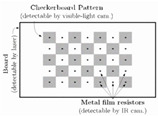	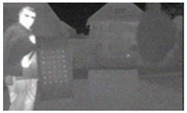
[48]—C	Printed black asymmetrical circular pattern on a white Dibond (aluminum composite) board	O—Sunlight	–	RMSE: 0.348	–	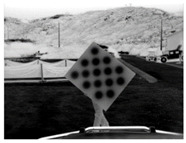
[6]—C	UV-printed symmetrical circular pattern on a Dibond (aluminum composite) panel	I—Electric fan heater	0.67/0.83	RMSE: 0.052	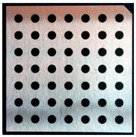	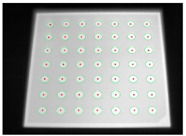
[49]—C	UV-printed symmetrical circular pattern on a Dibond (aluminum composite) panel	O—Sunlight	0.67/0.83	–	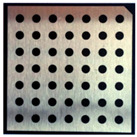	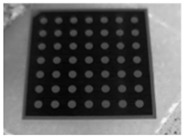
[50]—C	Anodized perforated Al-6061 symmetrical circular grid placed on a black coated base	I—Electric heater	0.04/0.95	–	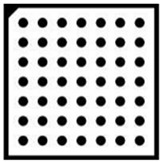	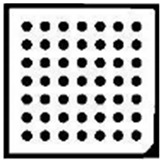
[51]—C	Black perforated symmetrical circular cardboard grid placed on top of a wooden plank wrapped in aluminum foil	I—N/A	-/0.94	–	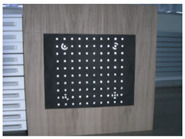	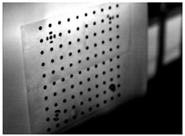
[52]—C	Laser-cut asymmetrical circular cardboard pattern	I—Heat gun	-/0.81	MRE: 0.500	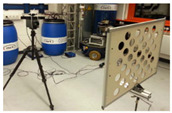	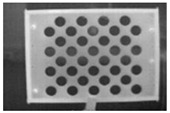
[53]—C	Laser-cut asymmetrical circular acrylic pattern	I—Heat gun	–	–	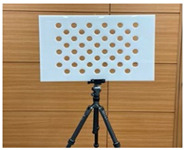	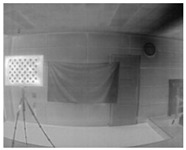

**Table 2:** Physical and thermal properties of the SS304 calibration plate.

	Properties	Values [65]
Physical properties	density (ρ)	$7900\;\mathrm{kg}/m^{3}$
Thermal properties	thermal conductivity (k)	16.3 W/m·K
	heat capacity $\left(c_{p}\right)$	500 J/kg·K
	linear thermal expansion $\left(\alpha_{L}\right)$	$16.6\times 10^{-6}\;\mathrm{mm}/\mathrm{mm}\cdot K\;@\;20-100\;K$
Target dimensions	length	150 mm
	width	136 mm
	thickness	6 mm

**Table 3:** Technical specifications of the thermographic camera used in this research.

Infrared Camera	Specifications
Model	InfraTec VarioCAM HD Head-600
Spectral range	7.5–14 μm
Temperature measuring range	−40 °C to +1200 °C
Temperature sensitivity	0.03 °C at +30 °C
Measurement accuracy	±1.5 °C
Detector	640×480 UFPA
Image framerate	60 Hz

**Table 4:** Emissivity coefficients (ε) of the calibration targets.

	Laser-Engraved	UV-Printed
Black squares	0.811	0.931
Bare metal squares	0.172	0.239

**Table 5:** List of reprojection errors (MRE and RMSE) for the two calibration targets.

	Laser-Engraved		UV-Printed	
Trials	MRE (Pixels)	RMSE (Pixels)	MRE (Pixels)	RMSE (Pixels)
1	0.1366	0.1623	0.0538	0.0602
2	0.1373	0.1638	0.0544	0.0605
3	0.1254	0.1500	0.0532	0.0591
4	0.1216	0.1453	0.0579	0.0646
5	0.1218	0.1456	0.0558	0.0622
6	0.1153	0.1373	0.0553	0.0617
7	0.1066	0.1265	0.0592	0.0661
8	0.1074	0.1277	0.0593	0.0660
9	0.0982	0.1172	0.0592	0.0662
Mean	0.1189	0.1417	0.0565	0.0630
Standard deviation	0.0134	0.0160	0.0025	0.0028

## Data Availability

Not applicable.
